# Supplementary material for: Comprehensive analysis of the microbiome in Apis cerana honey highlights honey as a potential source for the isolation of beneficial bacterial strains
Source: PeerJ. 2024 Mar 27;12:e17157. doi: 10.7717/peerj.17157 (PMC10981410; doi:10.7717/peerj.17157)
Supplement: Supplemental Information 1 [file peerj-12-17157-s001.docx]

**Table S1**. **Primer pairs were used to detect Nosema spp. and bee viruses.**

| **Primer** | **Sequence** | **Size (bp)** | **References** |
| --- | --- | --- | --- |
| *Nosema* spp.-F | 5’-CTGCCTGACGTAGACGCTAT-3’ | 593 | *ChaimaneeWarrit & Chantawannakul, 2010* |
| *Nosema* spp.-R | 5’-CTTCGATCCTCTAGCTTACG-3’ |  |  |
| ABPV-F | 5’-TTA TGT GTC CAG AGA CTG TAT CCA-3’ | 900 | *Benjeddou et al., 2001* |
| ABPV-R | 5’-GCT CCT ATT GCT CGG TTT TTC GGT-3’ |  |  |
| BQCV-F | 5’-TGG TCA GCT CCC ACT ACC TTA AAC-3’ | 700 | *Benjeddou et al., 2001* |
| BQCV-R | 5’-GCA ACA AGA AGA AAC GTA AAC CAC-3’ |  |  |
| CWV-F | 5’-GAT GAA CGT CGA CCT ATT GAA AAA GTT AAT C-3’ | 403 | *Thu et al., 2016* |
| CWV-R | 5’-GTG GGT TGG CTA TGA GTC ATC ATG TAT ACT G-3’ |  |  |
| DWV-F: | 5’-TCA TCT TCA ACT CGG CTT TCT ACG-3’ | 479 | *Yue & Genersch, 2005* |
| DWV-R: | 5’- CGA ATC ATT TTC ACG GGA CG-3’ |  |  |
| KBV-F | 5’-GAT GAA CGT CGA CCT ATT GA-3’ | 415 | *Stoltz et al., 1995* |
| KBV-R | 5’-TGT GGG TGG CTA TGA GTC A-3’ |  |  |
| IAPV-F | 5’-GAT TTG AGA GAT GTA TTT CCT TCT GCG G-3’ | 725 | *Thu et al., 2016* |
| IAPV-R | 5’-ACA CTT GCG TTG GTC CTG AAT GTT AAT GG-3’ |  |  |
| SBV-F | 5’- ACC AAC GAT TCC TCA GTA G-3’ | 487 | *Grabensteiner et al., 2001* |
| SBV-R | 5’- CCT TGG AAC TCT GCT GTG TA-3’ |  |  |
